# Two new West Palaearctic species of *Atelestus* Walker (Diptera, Atelestidae) and new distributional records of the family

**DOI:** 10.3897/zookeys.955.53698

**Published:** 2020-08-05

**Authors:** Liliana Kanavalová, Štěpán Kubík, Miroslav Barták

**Affiliations:** 1 Department of Zoology and Fisheries, Faculty of Agrobiology, Food and Natural Resources, Czech University of Life Sciences Prague, Kamýcká 129, 165 00 Praha-Suchdol, Czech Republic Czech University of Life Sciences Prague Czech Republic

**Keywords:** Atelestidae, *
Atelestus
*, descriptions, Diptera, Europe, *
Nemedina
*, taxonomy

## Abstract

*Atelestus turcicus* Barták, **sp. nov.** (Turkey) and *Atelestus ibericus* Barták, **sp. nov.** (Spain) are described and illustrated. A key to all known Palaearctic species of *Atelestus* is provided and the main diagnostic characters are discussed. The female of *Nemedina acutiformis* Carles-Tolrá, 2008 is described for the first time. New distributional records are presented: *Atelestus dissonans* Collin, 1961 – first records from Spain and Bulgaria, *A. pulicarius* (Fallén, 1816) – first record from Turkey, *Nemedina alamirabilis* Chandler, 1981 – first record from Bulgaria and *N. acutiformis* Carles-Tolrá, 2008 – first record from Turkey.

## Introduction

In the light of the unprecedented reduction in biodiversity and the possible further mass extinction of biota we should save species at least in collections for further studies (Barták 2019).

The family Atelestidae is a very small family of DipteraBrachycera, often assigned to the large superfamily Empidoidea. Numerous works have tried to elucidate phylogenetic relationships within Empidoidea including placement of the family Atelestidae. A comprehensive summary of empidoid morphology and relationships was provided by Sinclair and Cumming (2006). This key work was preceded or followed by a number of other important works. The above-mentioned classification was primarily proposed by Chvála (1983), but later molecular studies have clarified relationships further. The contribution by Wiegmann et al. (1993) was the first modern computer-generated analysis based on morphological characters to investigate the origins of the Cyclorrhapha. However, the first molecular study was provided by Collins and Wiegmann (2002), followed by Moulton and Wiegmann (2004, 2007). Recently, Wahlberg and Johanson (2018) studied phylogenetic relationships within Empidoidea based on mitochondrial and nuclear genes. All these molecular studies found the family Atelestidae to be sister group to the remaining Empidoidea. In addition, newly described amber fossil species have helped to clarify empidoid relationships (Grimaldi and Cumming 1999), in particular the work by Sinclair and Kirk-Spriggs (2010), which provides two new species for the family Atelestidae. The manuscripts from Chandler (1981), Sinclair and Arnaud (2001), Sinclair and Shamshev (2003), Sinclair and Papp (2004) and Carles-Tolrá (2008) significantly contributed to the knowledge of the genus *Nemedina*. These works above were complemented by relatively recent atelestid catalogs, checklists and faunistic records. For example, the work by Chvála (1983) provided an overview of all Nordic empidoid species. The same author created an atelestid checklist of Czech Republic and Slovakia (Chvála 2009), but his most important works are a Catalogue of Palaearctic Diptera, including family Atelestidae (Chvála 1989) and a list of valid European atelestid species on the „Fauna Europaea“ website (Chvála 2013). Further information about Afrotropical Atelestidae was submited by Sinclair (2017) in Manual of Afrotropical Diptera. Faunistic records of Atelestidae have also been published by country, e.g., from Germany with a contribution by Franzen (1996), from Bulgaria by Dzhambazov and Beschovski (2000), from French Corsica by Grootaert and Shamshev (2003), from Finland by Kahanpää (2014); from Hungary by Papp (2009), from Portugal by Andrade and Chandler (2016), and from Russia by Shamshev (2016) and Kustov et al. (2016).

The following species of extant Atelestidae are known from the West Palaearctic: three species of *Nemedina* Chandler (*N. alamirabilis* Chandler, 1981; *N. acutiformis* Carles-Tolrá, 2008 and *N. zaitsevi* Sinclair & Shamshev, 2003), two species of *Atelestus* Walker (*A. dissonans* Collin, 1961 and *A. pulicarius* (Fallén, 1816)) and one species of *Meghyperus* Loew (*M. sudeticus* Loew, 1850). Two additional species of *Atelestus* are added herein. The first assignment of the genus *Nemedina* to the family Atelestidae was proposed by Barták (2000) and this proposal was further supported and confirmed by later studies – Sinclair and Shamshev (2003), Sinclair and Papp (2004) and Sinclair and Cumming (2006).

## Material and methods

The material studied originated from recent collections of authors MB and ŠK in Spain and Turkey and it is deposited in the collection of the Czech University of Life Sciences, Prague (CULSP). The material was collected by means of mass trapping methods (sweeping vegetation, yellow and white water pan traps) and stored in ethyl alcohol. Voucher specimens were selected and dried using method described by Barták (1997).

Genitalia preparations and drawings: genitalia, together with the preceding 2 or 3 abdominal segments were removed from the rest of the body using small scissors and macerated in potassium hydroxide solution (approx. 10%) in small vials submerged in hot water for 1–2 hours. After neutralizing with 8% acetic acid (5 minutes), the genitalia were dissected in glycerine and photographed. The photos were produced using a Nikon SMZ 1500 stereomicroscope equipped with a Canon EOS 700D digital camera and were aligned and stacked using Adobe Photoshop. Images served as model for hand drawings, details were added directly observing objects.

The morphological terms used here follow Cumming et al. (1995), Merz and Haenni (2000), Sinclair (2000) and Sinclair and Cumming (2006). All body measurements (including body and setae length) were taken from dry specimens (therefore the actual length may differ from that of fresh or wet-preserved material) by means of an ocular micrometer mounted on Nikon SMZ 1500 binocular microscope. Male body length was measured from antennal base to the tip of genitalia and female body length from base of antennae to the tip of cerci. Thoracic setae are counted on one side of body.

## Taxonomy

### Descriptions of new species

#### Atelestus turcicus

Taxon classificationAnimaliaDipteraAtelestidae

Barták sp. nov.

87C8E20B-3B4E-5969-BA44-C5888FA39BD9

http://zoobank.org/FB1FCE1B-D431-477F-A6F1-F7C2ECCCC1B6

Figs 1 , 2

##### Type material.

***Holotype*** ♂, Turkey: Muğla, University campus, MT, 720 m, 37°09'42"N, 28°22'13"E, H. Kavak, 26.v.–26.vi.2015 (CULSP). ***Paratypes***: 7 ♂, 1 ♀, same data as holotype; 1 ♀, Turkey: 13 km NE of Muğla, pasture/pine wood, 1200 m, 37°14'50"N, 28°30'E, Barták, Kubík, 23.–27.vi.2015; 1 ♂, Turkey: Muğla, University campus, YPWT, 720 m, 37°09'42"N, 28°22'13"E, Barták, Kubík, 26.–27.vi.2015; 3 ♂, Turkey: Muğla, University campus, SW [= sweeping]+PT, 700 m, 37°09'42"N, 28°22'21"E, Barták, Kubík, 17.–22.v.2011; 2 ♂, same locality, O. Dursun, May 2013; 21 ♂, 4 ♀, Turkey: Muğla, University campus, MT, 720 m, 37°09'42"N, 28°22'13"E, H. Kavak, May 2015; 3 ♂, same locality, H. Pala, June 2016; 2 ♂, same locality, H. Pala, July 2016 – (CULSP).

**Figures 1, 2.**
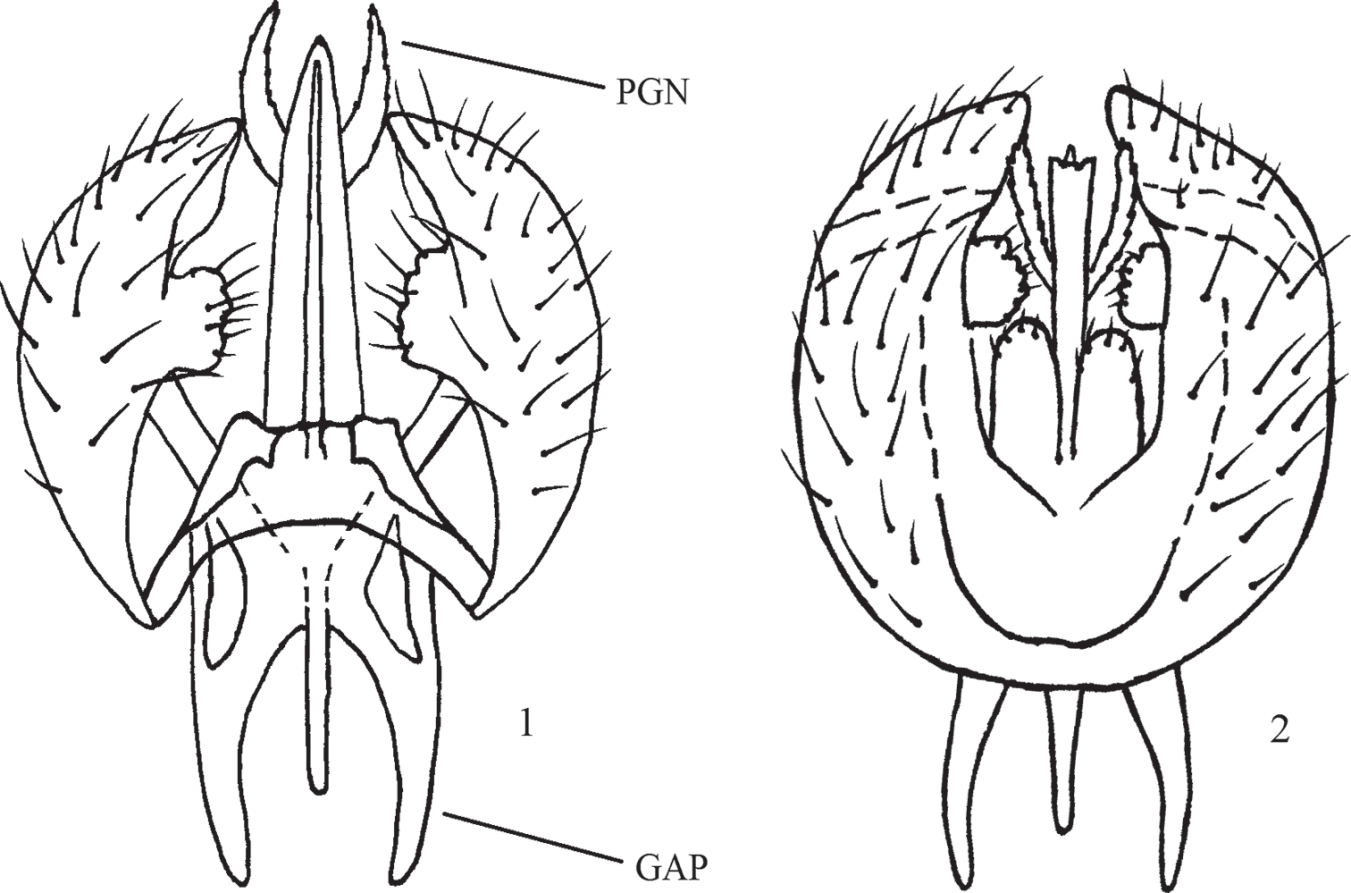
*Atelestus turcicus* sp. nov., male genitalia **1** ventral view **2** dorsal view. GAP = gonocoxal apodeme, PGN = postgonites.

##### Diagnosis.

Small brownish black, pale (white to yellow) setose species of *Atelestus*. Male genitalia: anterior part of gonocoxal apodeme short (at most 0.10 mm long), postgonites serrate, and hypandrium in ventral view nearly square-shaped, with long posterior lobes pointed upwards. Female frons shiny.

##### Etymology.

The species is named after country of origin (Turkey).

##### Description.

***Male head*** black, holoptic, entirely rather light grey microtrichose, white setose (except several dark setulae on labellum). Eye large, ventral third with smaller facets sharply divided with horizontal line from dorsal part with much larger facets. Occiput in dorsal part with row of short postocular setae (setae about as long as ocellar triangle), in lower part more densely short setose. Frons (very small triangle above antennae) without setae. Face including clypeus and gena microtrichose, without setae, postgena with several longer setae (about as long as ocellars). Ocellar setae short (about 0.15 mm). Palpus brown, slightly clavate, with several short setulae; labrum very short (about 0.08 mm), postmentum microtrichose, with several setae, labellum broad, covered with brownish setulae. Antenna black, pedicel with circlet of very short setulae (0.03 mm); postpedicel broadly pear-shaped; length of antennal segments (scape: pedicel: postpedicel: 1^st^ segment of stylus: stylus, in 0.01 mm scale) = 3: 4: 7–10: 1–2: 23–26. ***Thorax*** brownish black, sparsely grey microtrichose, without stripes, whitish to yellowish setose. Chaetotaxy: antepronotum with 1–3 rather long setulae, propleura with 1–2 similar setulae, proepisternum and prosternum without setae; postpronotum with 1 long seta and several additional much shorter setae; acrostichals biserial, setae about as long as distance between acrostichals and dorsocentrals (about 7–9 setae in one row); dorsocentrals irregularly biserial as long as acrostichals, inner row ending in 2 long prescutellars, outer row diverging and curved towards postpronotal lobe anteriorly; 1 presutural and 2 postsutural intraalars, 1 presutural supraalar; notopleuron with 3–4 strong and long setae; 1 postalar seta, 2 pairs of long scutellars, outer pair shorter. Laterotergite bare. ***Legs*** including coxae brownish black, microtrichose, all knees and bases of tibiae yellow to various extent (in darkest specimens basal third to fourth of fore and mid tibiae and basal fifth of hind tibiae pale and in lighter specimens up to half of fore and mid tibiae yellow and basitarsi partly yellowish brown), most setae and setulae pale, tarsi partly dark setulose. Fore femur short setulose, posteroventral row of setae nearly as long as femur depth, anteroventrals much shorter. Fore tibia narrow and without conspicuous setae, fore basitarsus with several elongate setulae ventrally. Mid femur short setose, with long preapical anteroventral seta and several posteroventrals on apical part. Mid tibia narrow, short setulose, with single rather long dark preapical ventral seta. Hind femur slightly widened, with rows of dorsal and anteroventral setae nearly as long as femur depth (longest dorsals situated on basal half and longest anteroventrals on apical third). Hind tibia ”club-shaped“, uniformly short setulose, hind basitarsus very slightly dilated. ***Wing*** clear, covered with microtrichia, Sc incomplete, stigma brown, radial veins brown, remaining veins yellowish, anal vein complete and depigmented, axillary angle slightly acute, C terminating at M_1+2_, basal costal seta pale and relatively short. Halter brownish black, calypter pale with short yellow fringes. ***Abdomen*** blackish brown, microtrichose, pale (yellow to white) setose. Lateral marginal setae on tergites nearly as long as segments; discal setae short, forming almost regular row, tergites 7 and 8 desclerotized and without setae. Hind marginal setae on segment 8 short; sternites with 5–8 pairs of pale setae, sternite 1 setose. Tergite 8 L-shaped as in other *Atelestus* spp., vertical narrow part bare, horizontal lower part broader and armed with 1–3 short setae, sternite 8 divided into two separate sclerites, each with 2–4 short setae. Genitalia as in Figs 1, 2: generally very similar to remaining three *Atelestus* spp., hypandrium short, roughly rectangular (lateral margins nearly parallel), desclerotized in middle part, with posterior hypandrial lobes directed upwards (similarly as in Fig. 9); epandrium ovoid, in posterior view ending with blunt tip. ***Length***: body 2.0–2.8 mm, wing 1.5–2.1 mm.

**Female.** Similar to male except the following: frons broad, entirely shiny including vertex, ocellar triangle and occiput microtrichose. Frons with submedian pair of setae subequally long and strong as verticals and pair of ocellars, ocellar triangle with additional two pairs of smaller setulae. Abdomen very short setose. Legs somewhat paler than in male, often also apex of coxae yellowish; hind tibia less distinctly clavate. Cercus long and slender. ***Length***: body 1.8–2.3 mm, wing 2.4–2.9 mm.

##### Remarks.

**Figures 3, 4.**
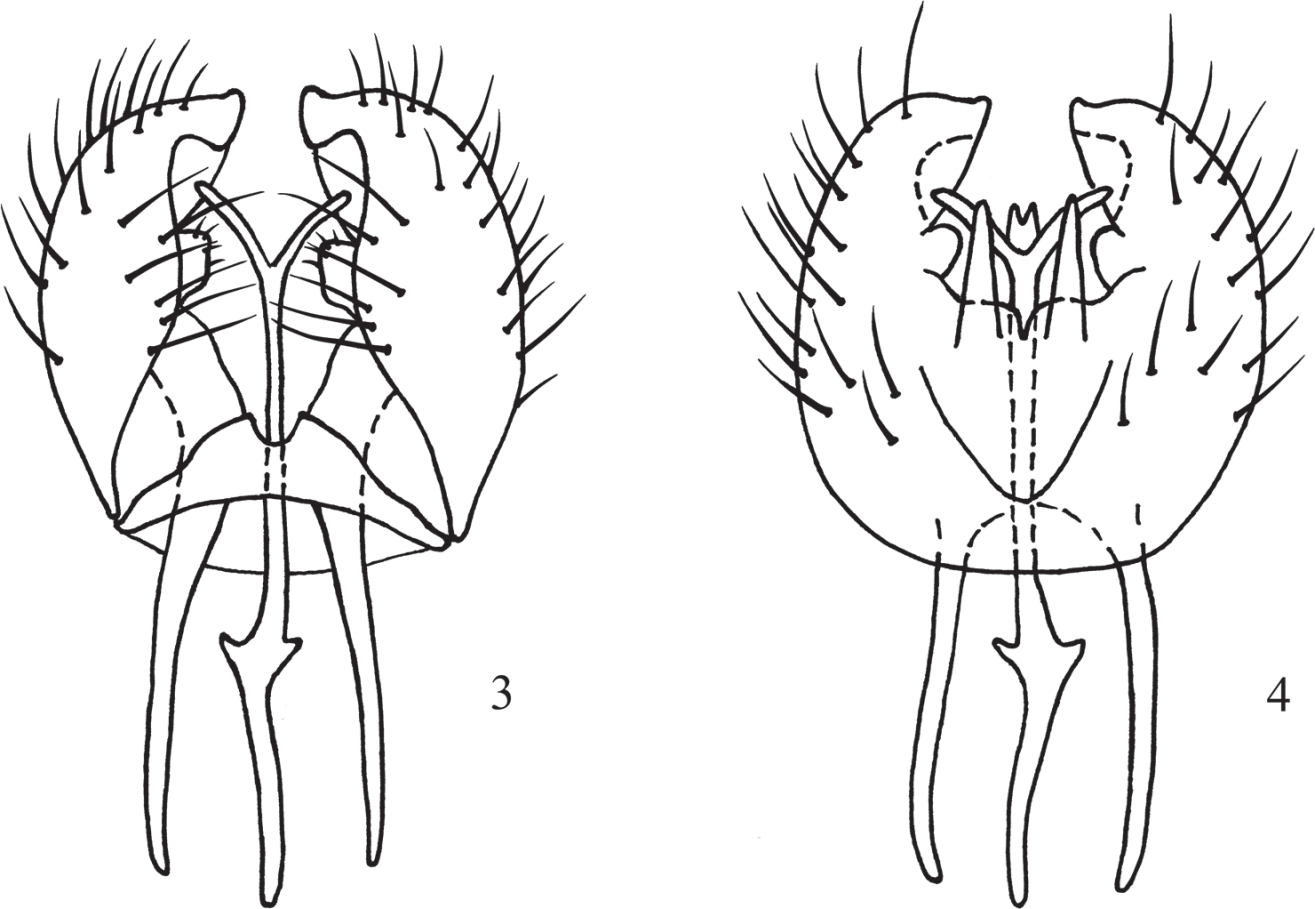
*Atelestus ibericus* sp. nov., male genitalia **3** ventral view **4** dorsal view.

The new species described above may be easily recognized by the entirely pale (white to yellow) setose body. It is rather similar to *A. ibericus* sp. nov. in having a small body and wing size, and a short setulose hind tibia. The shiny female frons is similar to *A. dissonans*, but this character remains unknown in female of *A. ibericus* sp. nov. There are several small differences in genitalia between three similar small species: *A. ibericus* sp. nov. has a long anterior part of the gonocoxal apodeme (about 0.20 mm long; Figs 3, 4) and unserrated postgonites. In both *A. turcicus* sp. nov. and *A. dissonans* the anterior part of the gonocoxal apodeme is short (at most 0.10 mm long) and the postgonites are serrated. The hypandrium in *A. dissonans* forms almost an equilateral triangle in ventral view with posterior lobes pointed upwards; in *A. turcicus* the hypandrium is nearly square-shaped in ventral view, with long posterior lobes pointed upwards; in *A. ibericus* sp. nov. the hypandrium is short triangle-shaped, without posterior lobes. Additionally, in *A. pulicarius* the hypandrium forms an elongated (isosceles) triangle with posterior lobes pointed posteriorly.

#### Atelestus ibericus

Taxon classificationAnimaliaDipteraAtelestidae

Barták sp. nov.

9FEF9D60-2B58-579D-AD6B-43EB4CAA5D02

http://zoobank.org/65A38384-DB02-4865-97B6-9B345AE2B8FF

Figs 3 , 4

##### Type material.

***Holotype*** ♂, Spain, Embalse de Barbate, SW [= sweeping], pasture nr. river, 37 m, 36°25'51"N, 5°44'38"E, Barták, Kubík, 6.–8.v.2017 (CULSP). ***Paratypes***: 3 ♂, Embalse de Barbate, SW, meadow + cork oak, 55 m, 36°24'10"N, 5°44'16"E, Barták, Kubík, 5.–9.v.2017; 1 ♂, 8 km NE from Alcalá de los Gazules, SW, oak forest, 335 m, 36°30'50"N, 5°39'50"E, Barták, Kubík, 7.–8.v.2017 – (CULSP).

##### Diagnosis.

Small brownish black species of *Atelestus* with most setae black but abdomen partly pale setose. Male with long anterior part of gonocoxal apodeme, postgonites not serrate, and hypandrium short triangle-shaped, without posterior lobes. Female remains unknow.

##### Etymology.

The species is named after the region of origin (Iberian Peninsula).

##### Description.

***Male head*** black, holoptic, entirely rather dark grey microtrichose, black setose. Eye large, ventral third with smaller facets sharply divided with horizontal line from dorsal part with larger facets. Occiput in dorsal part with row of medium long inclinate postocular setae (setae about as long as postpedicel), in lower part more densely medium moderately long setose, setae mostly black. Frons (very small triangle just above antennae) without setae. Face including clypeus and gena microtrichose, without setae, postgena with several longer pale setae (about as long as ocellars). Ocellar setae medium moderately long (about 0.20 mm), black, ocellar triangle with one pair of additional small posterior setae. Palpus brown, slightly clavate, with several short setulae; labrum very short (about 0.10 mm), postmentum microtrichose, with several setae, labellum broad, covered with black setulae. Antenna black, pedicel with circlet of short setulae (up to 0.09 mm); postpedicel broadly pear-shaped; length of antennal segments (scape: pedicel: postpedicel: 1^st^ segment of stylus: stylus, in 0.01 mm scale) = 3–4: 5–6: 8–9: 1–2: 25–29. ***Thorax*** brownish black, sparsely grey microtrichose, without stripes, mostly black setose. Chaetotaxy: antepronotum with 1 rather long pale lateral seta and row of 6–10 short pale setulae, propleura with 1 seta, proepisternum with 0–1 setae, prosternum without setae; postpronotum with 1 long seta and several additional much shorter setae; acrostichals biserial, setae slightly longer than distance between acrostichals and dorsocentrals (about 8–10 setae in one row); dorsocentrals irregularly 2–3-serial, as long as acrostichals, inner row ending in 2 long prescutellars, outer row slightly diverging outwards; 1 presutural and 2 postsutural intra-alars, 1 presutural supra-alar; notopleuron with 3 long setae; 1 postalar seta, 2 pairs of long scutellars, outer pair shorter. Laterotergite bare. ***Legs*** including coxae brownish black, microtrichose, all “knees” and bases of tibiae yellow to various extent (in darkest specimens only “knees” yellowish, and in lighter specimens up to third of fore and mid tibiae brownish yellow and hind basitarsus partly yellowish), most setae and setulae black, largest setae (all setae on coxae, posteroventrals on fore femur and both antero- and posteroventrals on hind femur partly or entirely pale – variable). Fore femur short setulose, posteroventral row of setae as long as femur depth or slightly longer, posterodorsals slightly shorter than femur depth, anteroventrals much shorter. Fore tibia narrow, without conspicuous setae; fore basitarsus with two subbasal and slightly elongate setulae ventrally. Mid femur short setose, with long preapical anteroventral seta and row of posteroventrals. Mid tibia narrow, short setulose, with single rather long dark preapical ventral seta. Hind femur slightly widened, with rows of dorsal and anteroventral setae nearly as long as femur depth (longest dorsals situated on basal half and longest anteroventrals on apical third). Hind tibia clavate (slightly wider than hind femur), uniformly short setulose; hind basitarsus very slightly dilated. ***Wing*** with slight brownish tinge, covered with microtrichia, Sc incomplete, stigma brown, radial veins yellowish brown, remaining veins brownish yellow, anal vein complete and depigmented, axillary angle slightly obtuse, C terminating at M_1+2_, basal costal seta black and long. Halter brownish black, calypter whitish with short pale fringes. ***Abdomen*** blackish brown, microtrichose, most setae pale (yellow to white), those on dorsal part of tergites, posterior three segments and sometimes posterior sternites black (variable). Lateral marginal setae on tergites nearly as long as corresponding segments; discal setae short, forming almost regular row. Hind marginal setae on segment 8 long; sternites with 5–8 pairs of pale long setae, sternite 1 setose. Genitalia as in Figs 3, 4: generally very similar to other *Atelestus* spp., hypandrium short, triangular, without processes; epandrium ovoid, in posterior view ending with small process. ***Length***: body 2.0–2.7 mm, wing 2.0–2.2 mm.

**Female.** unknown.

##### Remarks.

The new species described above is rather similar to *A. turcicus* sp. nov. in having a partly pale setose body, small body and wing size, and short setulose hind tibia. The main difference is the black setose thorax in *A. ibericus* sp. nov., but pale setose in *A. turcicus* sp. nov. See also remarks under *A. turcicus* sp. nov.

### Additions to described species

#### Nemedina acutiformis

Taxon classificationAnimaliaDipteraAtelestidae

Carles-Tolrá, 2008

AD50B428-A5DE-508A-8547-BA0DEE5232B4

##### Description.

**Female** (first description). Only characters different from description of the female of *Nemedina alamirabilis* as described by Chandler (1981) and Sinclair and Papp (2004) or not specified in those papers are mentioned here.

**Figures 5–7.**
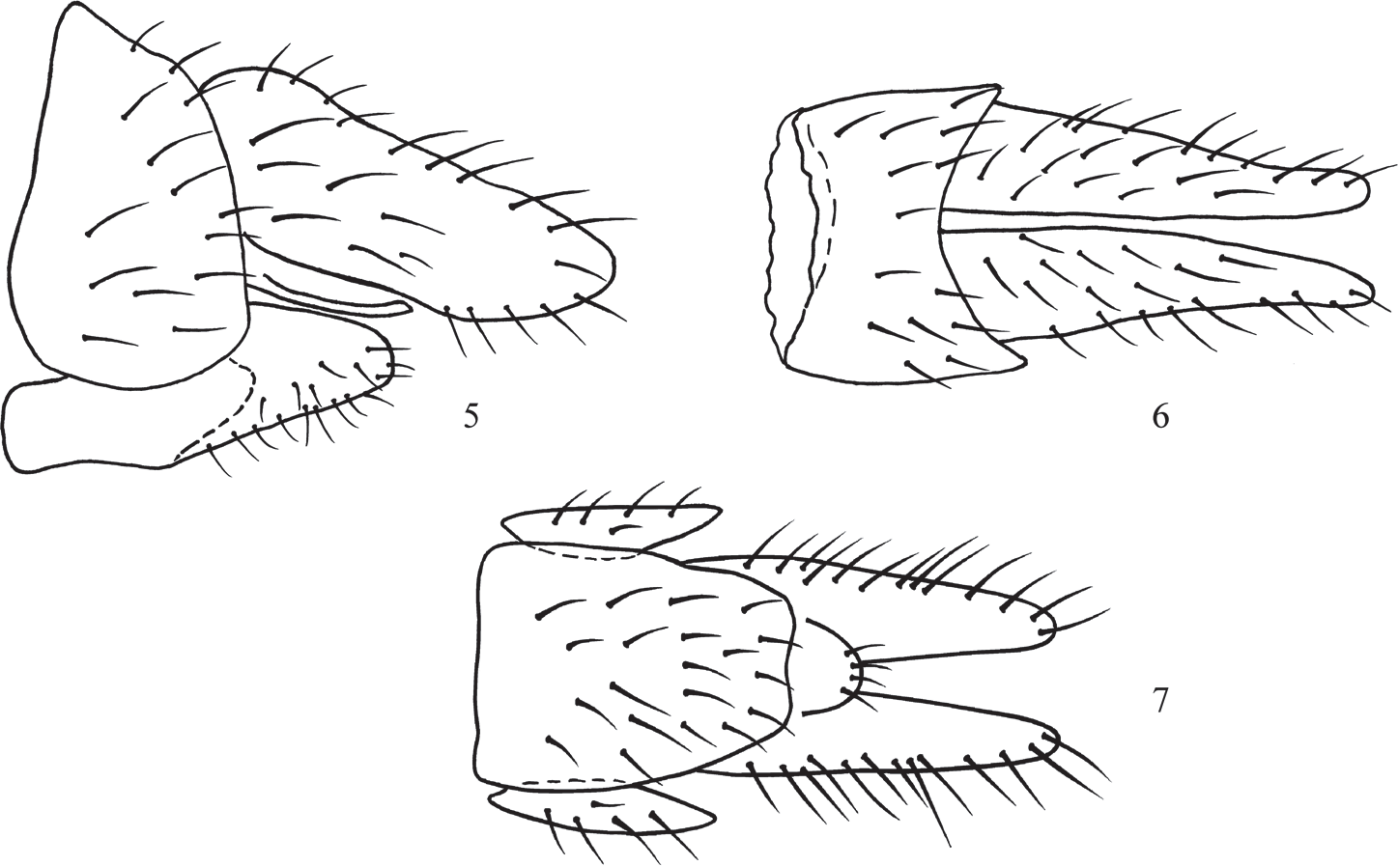
*Nemedina acutiformis* Carles-Tolrá, 2008, female abdomen **5** in lateral view **6** dorsal view **7** ventral view.

Eye with sparse and very short ommatrichia about as long as diameter of single facet. Frons broad, 0.14–0.15 mm at level of anterior ocellus, occupying 34–37% of head width, very sparsely microtrichose (best visible in dorsal view, frons almost subshiny in anterior view), covered with very short setulae (0.02–0.03 mm long) arranged in 3 irregular rows on each side (5–6 setulae in each row, outer rows continuing along eye margin up to vertex), leaving only narrow central area bare. Occiput and ocellar triangle covered with setulae about as long as those on frons. Gena medium wide (0.03 mm), lustrous. Thorax uniformly covered with short setulae and sparse microtrichiae. No apparent bare area between acrostichals and dorsocentrals. Notopleuron with 2–3 setae stronger, only slightly longer than surrounding setulae, postalar callus with 1 short seta, scutellum with 2–3 pairs of setae. Wing with anal vein almost complete and depigmented apically. Legs including coxae with very short setulae. Femora not compressed, fore tibia equally narrow. Hind tibia wider than hind femur. Abdomen microtrichose, only tergite 8 partly lustrous. Sternite 8 fully developed, hypoproct (sternite 10) present (Figs 5–7). Length of wing 1.3–1.5 mm.

##### Remarks.

Carles-Tolrá (2008) indicated two characters (beside genitalia) that distinguish males of *N. acutiformis* from *N. alamirabilis*: postpedicel ovate-conical and anal vein reaching wing margin. In fact both these characters are rather variable between our specimens of both species (males and females) and cannot be used as differentiating characters.

### Key to Palaearctic species of *Atelestus*

**Table:** 

1	Mesonotum with all setae pale (white to yellow)	***A. turcicus* sp. nov.**
–	Mesonotum with all setae black	**2**
2(1)	Abdomen with many setae pale (white to yellow)	***A. ibericus* sp. nov.**
–	Abdomen with all setae black	**3**
3(2)	Female frons microtrichose. Male mid tibia usually with several elongate dorsal setae; apex of epandrial lamella broadly ovate and microtrichose, hypandrium longer than wide in ventral view, with posterior lobes pointed posteriorly (L- to C- shaped in lateral view; Fig. 8); wing usually longer than 2.4 mm	***A. pulicarius***
–	Female frons shiny. Male mid tibia with all setae subequally long; apex of epandrial lamella narrowly ovate and shiny, hypandrium short (about as long as wide in ventral view), with posterior lobes pointed dorsally (U-shaped in lateral view; Fig. 9); wing usually shorter than 2.2 mm	***A. dissonans***

**Figures 8, 9.**
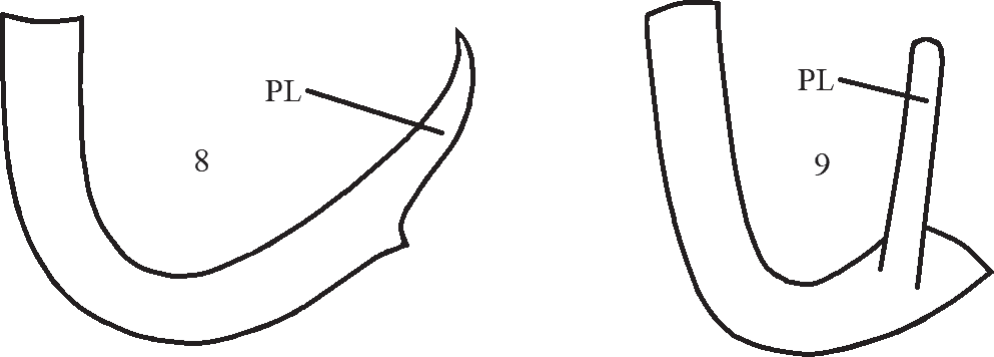
*Atelestus* species **8***A. pulicarius* (Fallén, 1816), hypandrium, lateral view **9***A. dissonans* Collin, 1961, hypandrium, lateral view. PL = posterior lobes.

### Faunistic records

#### Atelestus dissonans

Taxon classificationAnimaliaDipteraAtelestidae

Collin, 1961

44CB926E-C23D-5C5D-A58E-52BB92C4C5A0

##### Material examined.

3 ♂, Spain: Fragas Do Eume NP, MT, along brook, 60 m, 43°24'46"N, 8°03'50"E, Garcia and Ševčík, 20.vi.–19.vii.2019; 2 ♂, Bulgaria: Slanchev Brjag envir., PT, deciduous wood, 10 m, 42°45'03"N, 27°53'09"E, Barták, Kubík, 18.–20.vi.2019.

##### Remarks.

First records from Spain and Bulgaria. This species was reported from Portugal by Andrade and Chandler (2016).

#### Atelestus pulicarius

Taxon classificationAnimaliaDipteraAtelestidae

(Fallén, 1816)

9AB8B720-32A7-5BDA-8664-3DD4E5DF952A

##### Material examined.

4 ♂, 4 ♀, Turkey: 13km NE of Muğla, pine wood + pasture, 1100–1300 m, 37°15'N, 28°30'E, Barták, Kubík, 2.–3.v.2016.

##### Remarks.

First record from Turkey.

#### Nemedina alamirabilis

Taxon classificationAnimaliaDipteraAtelestidae

Chandler, 1981

157FF8FF-ACA0-5031-8DC9-E734E6EA89DA

##### Material examined.

2 ♂, Bulgaria: 2 km NE of Hristo Danovo, SW [= sweeping], forest path, 1160 m, 42°44'09"N, 24°37'15"E, Barták, Kubík, 15.vi.2017 (CULSP).

##### Remarks.

First record from Bulgaria.

#### Nemedina acutiformis

Taxon classificationAnimaliaDipteraAtelestidae

Carles-Tolrá, 2008

1F50B59F-5659-52C4-AA5E-73ED8F6F6310

##### Material examined.

11 ♂, 3 ♀, Turkey: Toparlar, lowland forest, SW, 36°58'39"N, 28°39'30"E, Barták, Kubík, 5.–7.v.2013; 1 ♂, Spain: Embalse de Barbate, SW, meadow + cork oak, 55 m, 36°24'10"N, 5°44'16"E, Barták, Kubík, 5.–9.v.2017 (CULSP).

##### Remarks.

First record from Turkey.

## Supplementary Material

XML Treatment for Atelestus turcicus

XML Treatment for Atelestus ibericus

XML Treatment for Nemedina acutiformis

XML Treatment for Atelestus dissonans

XML Treatment for Atelestus pulicarius

XML Treatment for Nemedina alamirabilis

XML Treatment for Nemedina acutiformis
